# The feasibility of small-caliber veins for autogenous arteriovenous fistula creation: A single-center retrospective study

**DOI:** 10.3389/fcvm.2023.1070084

**Published:** 2023-01-26

**Authors:** Ruijia Feng, Siwen Wang, Guangqi Chang, Wayne W. Zhang, Qinghua Liu, Xin Wang, Wei Chen, Shenming Wang

**Affiliations:** ^1^Department of Vascular Surgery, National-Guangdong Joint Engineering Laboratory for Diagnosis and Treatment of Vascular Diseases, The First Affiliated Hospital of Sun Yat-sen University, Guangzhou, China; ^2^Division of Vascular and Endovascular Surgery, Department of Surgery, University of Washington, Seattle, WA, United States; ^3^Department of Nephrology, The First Affiliated Hospital, NHC Key Laboratory of Nephrology, Guangdong Provincial Key Laboratory of Nephrology, Sun Yat-sen University, Guangzhou, China

**Keywords:** arteriovenous fistula, small-caliber vein, functional maturation, patency, tourniquet, ultrasound

## Abstract

**Objective:**

Autogenous arteriovenous fistula (AVF) is recommended as the first choice for hemodialysis vascular access. A small-caliber vein is one of the independent risk factors for AVF maturation and patency. However, the specific threshold is still unclear, making it difficult to accurately determine whether these vessels are suitable for AVF creation.

**Design:**

This is a single-center retrospective study.

**Method:**

Patients who underwent AVF creation in our medical center between January 2020 and September 2022 and satisfied the eligibility criteria were included in this retrospective study. Logistic regression analysis was performed to identify risk factors for functional maturation and additional intervention. The optimal cutoff value was determined based on the receiver operating curve (ROC) and the Youden index. Kaplan–Meier analysis was utilized in further patency rate comparisons.

**Result:**

A total of 125 forearm AVFs were created in 121 patients with end-stage renal disease (ESRD). The mean age was 53.88 ± 15.10  years. Preoperative vascular Doppler ultrasound (DUS) was conducted and recorded in 106 cases (84.80%). The mean targeted artery and vein diameters were 2.17 ± 0.54 and 1.71 ± 0.75  mm, respectively. Small-caliber vein is the risk factor for functional maturation failure (OR = 0.256, 95%CI [0.06–0.75], *p* = 0.033) and additional intervention (OR = 0.306, 95% CI [0.09–0.78], *p* = 0.031). The optimal cutoff value is 1.35  mm (augmented) when specificity and sensitivity reach 80 and 63.7%, respectively. The AVFs with a vein diameter of more than 1.35 mm (augmented) showed higher patency rates (*p* < 0.01).

**Conclusion:**

After comprehensive DUS evaluation, intraoperative hydrodilation, postoperative active exercise and intensive DUS detection, and application of balloon-assisted maturation, if necessary, using a vein more than 1.35 mm (augmented), could achieve satisfactory functional maturation and postoperative patency in AVF formation.

## Introduction

Hemodialysis is the lifeline for patients with end-stage renal disease (ESRD) to maintain the water, electrolyte, acid, and base balance and to discharge metabolic waste. The vascular access types consist of the central venous catheter (CVC), arteriovenous graft (AVG), and autogenous arteriovenous fistula (AVF) (1). Compared with CVC or AVG, the incidence of hematological infection, mortality, and cardiovascular events is all lower after AVF (2). In addition, the cost associated with AVF is extremely low (3). Moreover, the 2019 update to the Kidney Foundation’s Kidney Disease Outcomes Quality Initiative (KDOQI) Clinical Practice Guidelines for hemodialysis access also advocated that AVF is the first choice for patients who require long-term regular hemodialysis (4).

As the first choice, an AVF is usually created in the nondominant forearm because of the convenience of hemodialysis and the preservation of the vascular resources in the upper arm and proximal forearm (5). Radiocephalic arteriovenous fistula (RCAVF) is the most common vascular access. Regarding the surgical outcomes, the primary patency rate and functional maturation rate of AVF were approximately 60–70% (6, 7). Many previous studies have explored the potential risk factors for maturation failure, such as gender, diabetes, cardiovascular disease, artery diameter, vein diameter, and vein distensibility (8–10). A systematic review indicated that the optimal range of arterial and venous diameters for maximum performance was more than 2 mm, and using veins of less than 1.5 mm was not advised (11). Although small-caliber veins may affect the maturation and patency of AVF, the exact vein diameter threshold is still unclear, and most studies did not illustrate whether venous diameter was measured with a tourniquet. In this single-center retrospective study, we explored the effect of vein diameter and other vascular Doppler ultrasound (DUS) indicators on AVF maturation and patency. In addition, we also tried to determine a more reasonable cutoff value, aiming to provide more feasibility for AVF creation in small-caliber veins.

## Methods

### Patients

This retrospective study included patients who underwent AVF placement in the Department of Vascular Surgery of the First Affiliated Hospital of Sun Yat-sen University between January 2020 and September 2022. Patients were eligible if they satisfied the following criteria: (1) the patients were diagnosed with ESRD with various pathological types; (2) the patients met the indication for regular hemodialysis; (3) AVFs were constructed in the patient’s forearm with autogenous vessels; and (4) the patients were followed up postoperatively with DUS or physical examination. Ethics approval for this study was obtained from the ICE for Clinical Research and Animal Trial of the First Affiliated Hospital of Sun Yat-sen University. Informed consent was also acquired from the patients.

### Variables and definition

A predefined table was designed to record some basic characteristics such as age, gender, hypertension, diabetes, coronary artery disease, cerebrovascular disease, and peripheral artery disease. The surgical strategy and vascular DUS data were also completely recorded. Based on the targeted artery and vein, forearm AVFs were divided into RCAVF, radiobasilic arteriovenous fistula (RBAVF), radio-median arteriovenous fistula (RMAVF), and ulnar-median arteriovenous fistula (UMAVF). Vascular DUS was conducted by professional vascular surgeons with a tourniquet tied around the proximal one-third of the upper arm. The diameter, peak systolic velocity (PSV) and flow volume (FV) of bilateral vessels, and anastomotic site conditions were evaluated both preoperatively and postoperatively for further analysis. The primary outcome was functional maturation, while the secondary outcomes included primary patency, assisted primary patency, secondary patency, and additional intervention. In accordance with the recommendation standard from the Society for Vascular Surgery (12), functional maturation was defined as a fistula became suitable for providing prescribed dialysis with two needles and able to deliver a flow rate of 350 to 400 ml/min without access recirculation to maintain a treatment time less than 4 h. Primary patency was defined as the duration of time from fistula placement to any intervention designed to maintain or reestablish patency, access thrombosis, or the time of measurement of patency, while assisted primary patency indicated the interval from the time of access placement until access thrombosis, stenosis, or the time of measurement of patency, including intervening manipulations designed to maintain the functionality of access. The secondary patency was defined as the duration time of intra-access patency that starts from the date of vascular access creation to vascular access abandonment, thrombosis, or the time of patency measurement including interventions (surgical or endovascular) designed to reestablish functionality in vascular access. The additional intervention included proximal new access creation and percutaneous angioplasty (PTA). Percutaneous angioplasty (PTA) performed before maturation was also called balloon-assisted maturation (BAM).

### Surgical strategy

**Figure 1:**
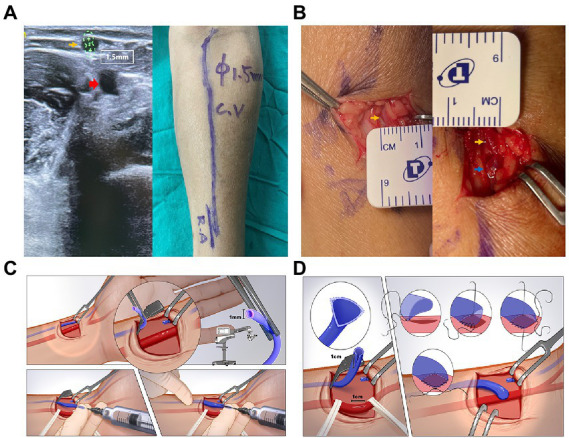
**(A)** Preoperative vascular ultrasound image (left) and whole-course vessels marking (right) of the distal left forearm. The vessel diameter was measured with a tourniquet. The yellow arrow indicates the cephalic vein, which is around 1.5 mm. The red arrow indicates the radial artery. **(B)** Cephalic vein measurement before (left) and after (right) end-to-side anastomosis. The yellow arrow indicates the cephalic vein and the blue arrow indicates the anastomotic site. **(C)** Detailed schematic diagram of the vein hydro-dilation stage. **(D)** Detailed schematic diagram of the fistula anastomosis stage.

After completing the Allen test (used for assessing palmar arch patency) and all relevant preoperative examinations, the patients underwent preoperative vascular DUS with a tourniquet tied around the proximal one-third of the upper arm. During the DUS examination, the diameter, PSV, FV, and intra-lumen condition of the bilateral vein and artery were considered comprehensively to evaluate the feasibility of AVF creation by vascular surgeons. Under the guidance of the ultrasonic probe and pulse palpation, we marked the whole vein and the location of the artery on the forearm (Figure 1A). The nondominated distal forearm was the most appropriate location of the AVF if there was no contradiction. After local infiltration anesthesia, we made an incision approximately 3 cm in length in the distal forearm. We separated the subcutaneous tissue to expose the targeted artery and vein (Figure 1B). After vein ligation and transection, we performed a segmental hydrodilation of the proximal segment of the vein as well as the whole length of the vein along with the forearm by finger pressing. If there were mural/fibrin thrombi or calcified plaques, we need to remove them before hydrodilation. Heparin saline was injected from the distal end of the vein through a blunt needle, while the fingers of the assistant alternatively moved along the preoperative marked pathway with conducting vein compression and release (Figure 1C). Then, a 7–10 mm (depending on the diameter of the vein) incision was made on the surface of the artery, and an end-to-side anastomosis was performed between the vein and the artery by running sutures with Prolene 8-0. The suturing detail is demonstrated in Figure 1D. Finally, we could palpate or auscultate the thrill on the surface of the fistula after a cosmetic skin suture. In approximately 2 weeks after AVF creation, postoperative DUS examination and fistula detection were used to assess AVF functional maturation for the first time.

### Statistical analysis

Continuous variables are expressed as mean ± standard deviation (SD) and categorical variables are shown as numbers and proportions. Student’s *t*-test and Mann–Whitney U test were used to compare continuous variables. The chi-square test and Fisher’s exact test were utilized to analyze the categorized variables. We conducted a logistic regression analysis to identify some risk factors for specific treatment outcomes (13). The odds ratios (ORs), 95% confidence intervals (CIs), and *p*-values were calculated subsequently. The optimal cutoff value was determined based on the receiver operating curve (ROC) and the Youden index (14). The predictive value was evaluated by the area under the receiver operating curve (AUC), which ranged from 0.5 to 1. AUC > 0.7 usually indicated good discrimination (15). Kaplan–Meier survival curves and log-rank tests were used to compare postoperative patency. All statistical analyses were performed by R software (version 4.1.1; https://www.r-project.org), with the pROC, survival, and survminer R packages. A two-sided *p*-value less than 0.05 indicated a statistically significant difference.

## Results

From January 2020 to September 2022, a total of 125 forearm AVFs were created in 121 patients with ESRD (Table 1). The mean age was 53.88 ± 15.10 years among 75 men (61.98%) and 46 women (38.02%). Among them, 111 patients (91.74%) had hypertension, 41 patients (33.88%) had diabetes, 15 patients (12.40%) had coronary artery disease, eight patients (6.61%) had cerebrovascular disease, and eight patients (6.61%) had peripheral artery disease. Most of the AVFs (94.40%) were created in the nondominant forearm. A total of 117 cases (93.60%) underwent RCAVF, while RBAVF, RMAVF, and UMAVF were created in four, two, and two cases, respectively. Preoperative vascular DUS was conducted and recorded in 106 cases (84.80%). For the other 19 cases, vascular DUS was completed in the operating room to assess vascular conditions, but the data were not fully recorded. The mean targeted artery diameter was 2.17 ± 0.54 mm (range 1.10–3.90 mm), while the mean targeted vein diameter was 1.71 ± 0.75 mm (range 0.70–4.10 mm). There was no significant difference in vein diameter between women and men (*p* = 0.62). The data representing vessel diameter distribution are shown in Supplementary Table S1. Other detailed DUS data of targeted vessels and brachial artery inflow tract are interpreted in Table 1.

**Table 1:** Patients’ demographic characteristics, ultrasound data, and surgical strategy.

Variable	Number (%)
Patients	121
Male	75 (61.98%)
Female	46 (38.02%)
Mean age (years)	53.88 ± 15.10
Hypertension	111 (91.74%)
Diabetes	41 (33.88%)
Coronary artery disease	15 (12.40%)
Cerebrovascular disease^a^	8 (6.61%)
Peripheral artery disease^b^	8 (6.61%)
Cases	125
Left arm	118 (94.40%)
Right arm	7 (5.60%)
RCAVF	117 (93.60%)
RBAVF	4 (3.20%)
RMAVF	2 (1.60%)
UMAVF	2 (1.60%)
Preoperative ultrasound	106 (84.80%)
Mean forearm artery diameter (mm)	2.17 ± 0.54
Mean forearm artery PSV (cm/s)	63.80 ± 64.10
Mean forearm vein diameter (mm)	1.71 ± 0.75
Mean forearm vein PSV (cm/s)	12.53 ± 4.95
Mean brachial artery diameter (mm)	4.36 ± 0.78
Mean brachial artery PSV (cm/s)	69.19 ± 21.36
Mean brachial artery FV (ml/min)	70.29 ± 48.91

RCAVF: radio-cephalic arteriovenous fistula; RBAVF: radio-basilic arteriovenous fistula; RMAVF: radio-median arteriovenous fistula; UMAVF: ulnar-median arteriovenous fistula; PSV: peak systolic velocity; FV: flow volume. Values are presented as mean ± SD or number and percentage. *p* < 0.05 was considered statistical significance.

a Cerebrovascular diseases include cerebral infarction, cerebral hemorrhage, transient ischemic attack, and cerebral arteriosclerosis.

b Peripheral artery diseases include atherosclerosis obliterans, thromboangitis obliterans, and arteriosclerosis.

**Figure 2:**
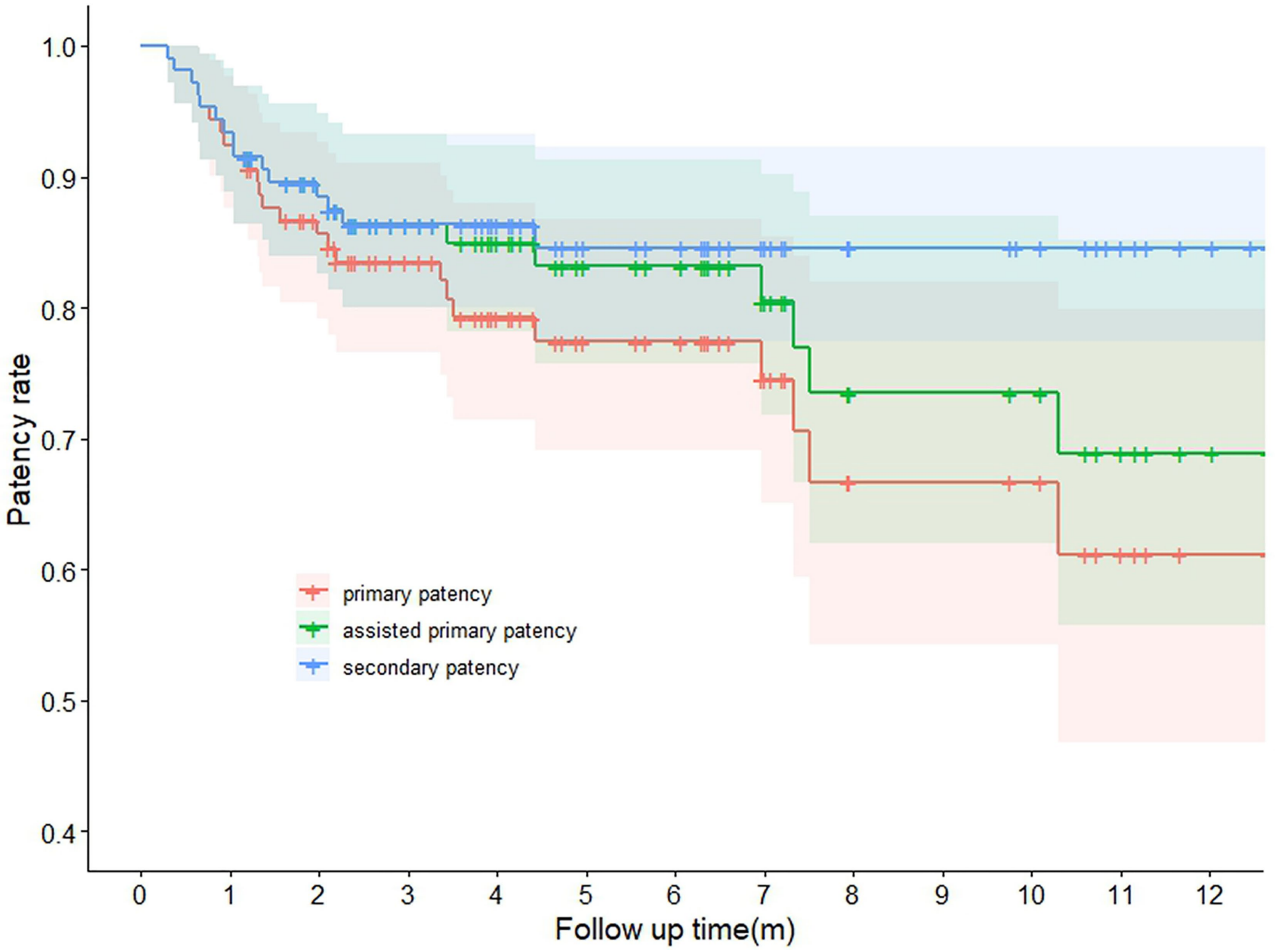
Postoperative patency rates of the 125 arteriovenous fistulas in long-term follow-up.

A total of 109 cases (87.20%) achieved functional maturation at the last follow-up with a mean maturation time of 66.22 days. The mean follow-up time was 204.54 days (Table 2). Among 16 failed cases, 12 cases developed acute thrombosis, which almost completely blocked the flow of the fistula and four of them had to have another AVF created in a more proximal site of the forearm. Four cases suffered from stenosis and inadequate FV and three of them conducted PTA. Acute thrombosis all formed within 1 month with a mean time of 20 days, while most stenosis cases were detected later within 2 months. Regarding mature cases, 12 cases underwent PTA and seven of them were performed as BAM to assist functional maturation. The postoperative patency is shown in Figure 2.

**Table 2:** Follow-up outcomes of the arteriovenous fistulas.

Variable	Number (%)
Total cases	125
Mean follow-up time (day)	204.54 ± 180.23
Functional maturation	109 (87.20%)
Mean maturation time (day)	66.22 ± 27.13
Functional maturation failure	16 (12.80%)
Acute thrombosis	12 (9.60%)
Stenosis and low flow volume	4 (3.20%)
Additional intervention	21 (16.80%)
Proximal new access creation	6 (4.80%)
Percutaneous angioplasty	15 (12.00%)

Values are presented as mean ± SD, number or percentage.

**Figure 3:**
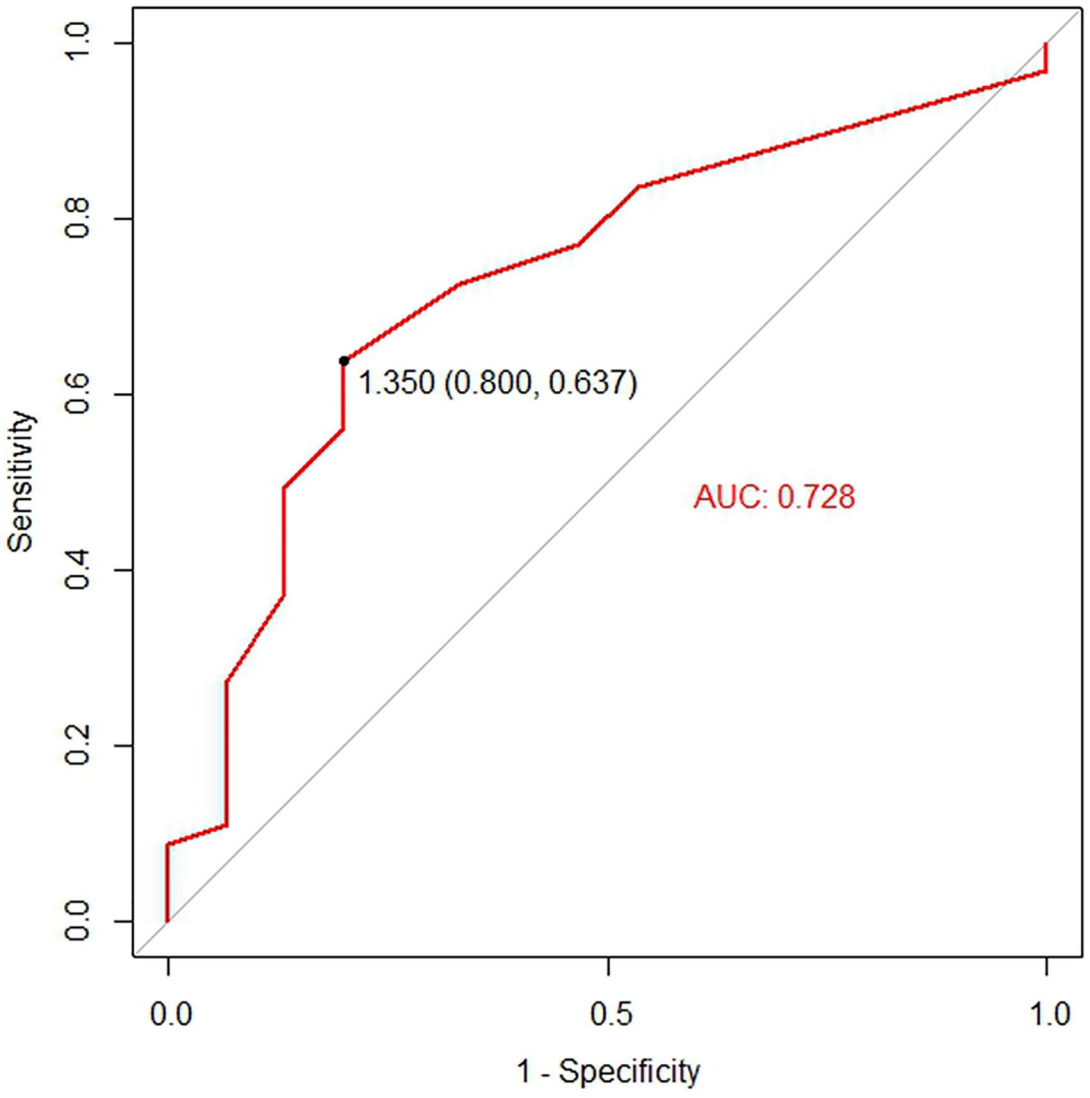
The receiver operating characteristic (ROC) analysis for the predictive value of vein diameter for functional maturation of the arteriovenous fistula (AVF). The black point on the ROC curve indicates the cutoff vein diameter at which optimal sensitivity and specificity were reached.

**Figure 4:**
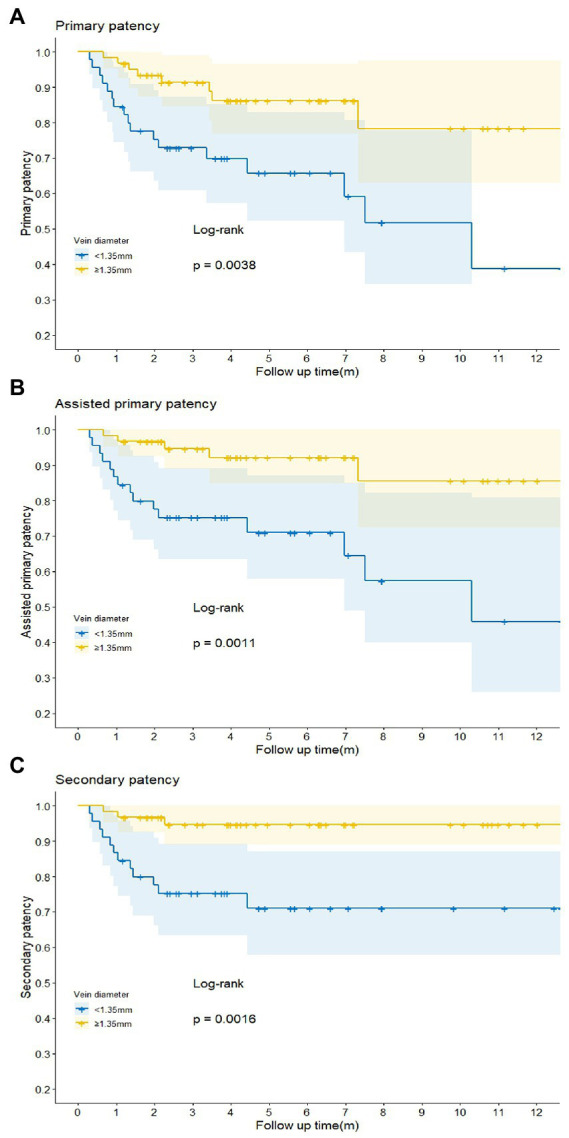
Comparison of the primary **(A)**, assisted primary **(B)**, and secondary **(C)** patency rates of the arteriovenous fistula (AVF) in different groups when vein diameter cutoff value was set at 1.35 mm. *p* < 0.05 was considered statistically significant.

Logistic regression analysis was utilized to explore the risk factors for functional maturation failure. Coronary artery disease (OR = 8.000, 95%CI [2.17–29.79], *p* = 0.002) and small vein diameter (OR = 0.256, 95%CI [0.06–0.75], *p* = 0.033) were significant risk factors (Table 3). There was no significant difference in gender, age, diabetes, artery diameter, or the brachial artery condition. The ROC curve showed the predictive value of the vein diameter for AVF maturation with AUC equal to 0.728. The optimal cutoff was 1.35 mm, with specificity and sensitivity of 80 and 63.7%, respectively (Figure 3; Supplementary Table S2). Then, we stratified the cases based on the cutoff value of vein diameter and evaluated the postoperative patency. Veins with a diameter of more than 1.35 mm showed higher primary, assisted primary, and secondary patency rates (*p* < 0.01; Figure 4). Patients with veins larger than 2 mm had better primary patency (*p* < 0.05). Surprisingly, there was no significant difference in assisted primary and secondary patency rates with 2 mm as the grouping threshold (*p* > 0.05; Supplementary Figure S1), and no significant difference exists in functional maturation as well (*p* > 0.05). In addition, if we grouped the patients based on maturation time, there was no significant difference in vein diameter in the different groups (Supplementary Figure S2). Another logistic regression analysis was performed to identify the risk factors for additional intervention (Supplementary Table S3). Small preoperative vein diameter (OR = 0.306, 95%CI [0.09–0.78], *p* = 0.031) also increased the incidence of additional intervention.

**Table 3:** Logistic regression analysis of risk factors for functional maturation failure in 106 cases with preoperative ultrasound data.

Variable	Functional maturation	Functional maturation failure	OR	95%CI	*p* value
Male	56/91	8/15	1.400	0.45–4.24	0.548
Female	35/91	7/15			
Mean age	51.75 ± 15.15	59.67 ± 18.29	1.033	0.99–1.07	0.080
Diabetes	28/91	7/15	1.969	0.63–6.02	0.231
Coronary artery disease	7/91	6/15	8.000	2.17–29.79	0.002
Cerebrovascular disease^a^	6/91	2/15	2.179	0.30–10.70	0.370
Mean forearm artery diameter (mm)	2.16 ± 0.55	2.19 ± 0.50	1.104	0.39–2.88	0.845
Mean forearm artery PSV (cm/s)	70.91 ± 89.73	63.43 ± 25.85	1.000	0.97–1.01	0.987
Mean forearm vein diameter (mm)	1.78 ± 0.76	1.31 ± 0.52	0.256	0.06–0.75	0.033
Mean brachial artery diameter (mm)	4.37 ± 0.80	4.29 ± 0.62	0.883	0.43–1.77	0.729
Mean brachial artery PSV (cm/s)	68.51 ± 20.73	73.18 ± 24.36	1.010	0.99–1.04	0.435
Mean brachial artery FV (ml/min)	67.46 ± 45.85	85.65 ± 60.72	1.006	0.99–1.02	0.214

OR: odd ratio; CI: confidence interval; PSV: peak systolic velocity; FV: flow volume. Values are presented as mean ± SD or number. *p* < 0.05 was considered statistical significance.

a Cerebrovascular diseases include cerebral infarction, cerebral hemorrhage, transient ischemic attack, and cerebral arteriosclerosis.

## Discussion

**Figure 5:**
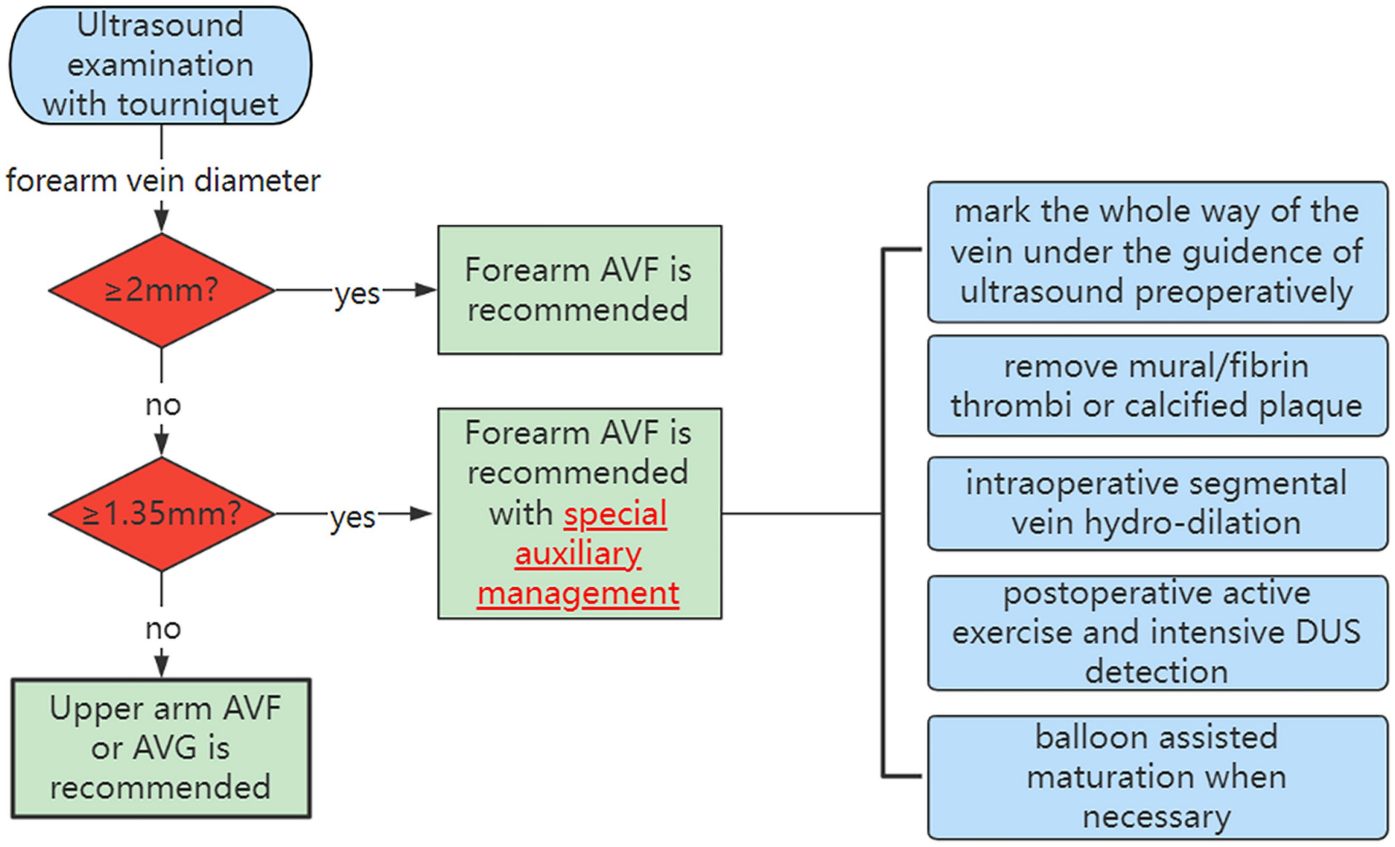
The strategies of access placement and maintenance for small-caliber veins.

In our study, the rate of functional maturation (87.20%) was much higher than that observed in some previous studies (6, 16, 17). The detailed and precise preoperative vascular DUS evaluation with a tourniquet contributes to an increased functional maturation rate (18, 19). Apart from the venous diameter, the distensibility of the vein also has a great influence on the long-term effect of AVF (10, 20). After AVF creation, arterial blood flows into the vein with high pressure and velocity. Veins with better compliance could expand better in terms of arterial blood pressure so that the local pressure on the venous wall can be balanced to avoid intima damage and further reduce the incidence of thrombosis or stenosis (21). Therefore, it is essential to tie the tourniquet in the upper arm when measuring the venous diameter, PSV, and FV by vascular DUS. In addition, proficient and experienced surgical techniques are also beneficial to obtaining more satisfactory outcomes. But the most important influencing factor for the functional AVFs created with small-caliber veins is the detailed strategy for creating and maintaining the functionality of access. It is not described comprehensively before. We summarized the strategies of access placement and maintenance for small-caliber veins (Figure 5) based on our results and the standard recommendation from the guideline (4).

In the access placement, with the assistance of DUS, we first checked whether there was mural/fibrin thrombus or calcified plaque in the vascular lumen, which need to be removed first. They cause turbulence and PSV change in AVF and further can cause neointima hyperproliferation, fistula stenosis, or even occlusion (22). Then we used a blunt needle and heparin saline for segmental dilation along with the target vein. The elasticity of the vein is not as good as that of the artery, and the diameter of the vein could be increased 2–3 times after repeated hydrodilation. As for vascular anastomosis skills, we chose end-to-side anastomoses using two running Prolene 8-0 sutures from both sides to the middle. The number of sutures should ensure tight anastomosis and reduce the occurrence of anastomotic site stenosis. Moreover, we taught patients how to exercise their fingers and palms regularly to increase fistula blood flow and promote AVF maturation. Patients were advised not to compress the forearm post-operatively and not to lift heavy objects with the operated arm for protecting the AVF, especially in the early postoperative period.

A total of 16 patients failed the AVF functional maturation due to acute thrombosis or fistula stenosis. For patients with acute thrombosis, we usually choose to establish another vascular access, rarely using thrombolysis or PTA. The reason is that it usually exceeded 7 days since thrombosis forms when the patients come back to the clinic. Besides red blood cells, platelets and fibrin are involved in the thrombus, and the texture becomes resistant. Meanwhile, the efficiency of thrombolysis or thrombectomy is not satisfactory (23). For patients with an anastomotic site, outflow vein, or inflow artery stenosis, various types of balloons are utilized for fistula dilation before or after maturation. This procedure is safe and effective immediately, which has become the standard management of AVF stenosis (24). Whether additional intervention is necessary may be related to the results of postoperative DUS (25). Postoperative DUS follow-up may become the critical strategy for maintaining the functionality of access, especially in the early 2 weeks. If DUS indicates anastomotic stenosis, low anastomotic PSV, small forearm vessels diameter, small brachial artery diameter, low brachial artery PSV, or FV, additional intervention such as BAM is considered to improve the functional maturation and long-term patency rates. The results are partially identical to the previous study (26).

Both primary balloon angioplasty (PBA) and BAM are manipulations of assisted maturation. Primary balloon angioplasty (PBA) is a technique of intraoperative dilation for small veins. In our result, we applied segmental hydro-dilation for all veins before anastomosis and the rate of functional maturation and primary patency was ideal. However, Pierfrancesco Veroux et al. (27) reported a comparative result between PBA and hydro-dilation in AVFs, which demonstrated that PBA of small (≤2 mm) cephalic veins improves the primary patency of AVF and decreases reintervention rate. We agreed with the efficiency of PBA and found that segmental hydro-dilation might leave some non-dilated sites along with the whole vein, which could lead to postoperative stenosis. Due to the expensive cost of the balloon and post-operative auxiliary management such as BAM and intensive DUS detection for patients with venous stenosis after segmental hydro-dilation, we still suggest that segmental hydro-dilation is a better strategy for creating AVF with a small vein. For BAM in the maintenance of AVF with small veins, a previous study proved that BAM could be used to improve the maturation rate of AVF with small veins, which is similar to our result (28).

The small-caliber vein is one of the independent risk factors for AVF functional failure and additional intervention, which is consistent with the conclusions of previous studies (29, 30). There has been no unified definition of the cutoff value of vein diameter and more studies chose 2 mm as the threshold for small-caliber veins (31–34). The KDOQI guidelines (4) also mentioned 2 mm when instructing the diameter of vein for constructing AVF. But It is suggested that veins smaller than 2 mm need careful and comprehensive evaluation before operation. Kordzadeh et al. (35) reported a study on the impact of patient demographics, anatomy, comorbidities, and peri-operative planning on the primary functional maturation of autogenous RCAVF. According to their result, a cephalic vein diameter of >1.55 mm (non-augmented) was found to be independently associated with the primary functional maturation of 86% in the RCAVF formation. In our study, all patients conducted vascular DUS with a tourniquet, and the diameter represented augmented vessels in which we take vein distensibility into consideration. According to the ROC curve, we used 1.35 mm (augmented) as the optimal cutoff value, which is much smaller than 1.5 mm (non-augmented) to further group patients and compare patency rates. We verified that veins of less than 1.35 mm negatively affect postoperative patency. To explore the predictive value of other thresholds, we used 2 mm (augmented) as the threshold and evaluated the influence on outcomes. The results indicate that there is no significant difference in assisted primary and cumulative patency rates, even though the primary patency is significantly different due to the absence of auxiliary management such as BAM, which may support improving the postoperative patency of small veins. Veins of less than 2 mm (augmented) should not be absolutely contraindicated for AVF creation, although there exists a significant difference in primary patency. Therefore, patients with a vein diameter of less than 2 mm have a risk of additional intervention after the establishment of AVF, thus, it is necessary to follow up more closely through ultrasound. As for the maturation time, the bar plot reflects that the vein diameter did not affect the AVF maturation time, which is not consistent with a previous study (36). The reason is that intraoperative segmental hydrodilation and regular postoperative follow-up shorten the maturation time of small-caliber vein AVF. Discrepancies in maturation time may be associated with gender and underlying diseases such as diabetes (37). Above all, we raise a flow diagram of appropriate strategies for AVF placement and maintenance of decisions and provide advice for patients with different vein diameters (Figure 5).

There are some limitations in our study. First, this was an observational and retrospective study. Second, preoperative DUS was performed by several different professors and the consistency of data measurement is difficult to guarantee, and not all patients were reexamined in our hospital after vascular access creation, thus, some of the DUS data were lost. Finally, postoperative active exercise and fistula protection need patients’ initiative cooperation, which also affects AVF maturation.

## Conclusion

The small-caliber vein is an independent risk factor for AVF maturation failure and additional intervention. Along with obtaining a comprehensive DUS evaluation, intraoperative segmental hydrodilation, postoperative active exercise, intensive DUS detection, and application of BAM if necessary, using a vein measuring more than 1.35 mm (augmented), could achieve satisfactory functional maturation and postoperative patency in AVF formation. This will provide more opportunities for patients with small-caliber veins for long-term hemodialysis.

## Data availability statement

The raw data supporting the conclusions of this article will be made available by the authors, without undue reservation.

## Ethics statement

The studies involving human participants were reviewed and approved by ICE for Clinical Research and Animal Trial of the First Affiliated Hospital of Sun Yat-sen University. The patients/participants provided their written informed consent to participate in this study.

## Author contributions

WC and SMW had full access to all of the data in the study and take responsibility for the integrity of the data and the accuracy of the data analysis, critical revision of the manuscript for important intellectual content, and supervision. RF, SWW, WC, and SMW: concept and design. RF, SWW, GC, WZ, QL, XW, WC, and SMW: acquisition, analysis, or interpretation of data, and administrative, technical, or material support. RF and SWW: drafting of the manuscript. RF, SWW, GC, WZ, QL, and XW: statistical analysis. SWW and SMW: obtained funding. All authors contributed to the article and approved the submitted version.

## Funding

This study was supported by the National Natural Science Foundation of China (Grant Nos. 81270378, 81070258, and 81370368), the Guangdong Province Industry-Academia-Research Program (2011B090400117), Guangzhou Science and Technology Plan Projects (2011Y2-00022), Guangdong Department of Science & Technology Translational Medicine Center grant (2011A080300002), and Natural Science Foundation of Guangdong Province (S2013040012593).

## Conflict of interest

The authors declare that the research was conducted in the absence of any commercial or financial relationships that could be construed as a potential conflict of interest.

## Publisher’s note

All claims expressed in this article are solely those of the authors and do not necessarily represent those of their affiliated organizations, or those of the publisher, the editors and the reviewers. Any product that may be evaluated in this article, or claim that may be made by its manufacturer, is not guaranteed or endorsed by the publisher.

## Supplementary material

SUPPLEMENTARY FIGURE 1 Comparison of the primary **(A)**, assisted primary **(B)** and secondary **(C)** patency rates of the arteriovenous fistula (AVF) in different groups when vein diameter cut-off value was set at 2mm. *P* < 0.05 was considered statistical significance.

SUPPLEMENTARY FIGURE 2 The different vein diameter distribution grouped by maturation time. Data are expressed as the mean ± SD and compared by two-tailed student’s t test between each two groups. The scattered points represented the specific vein diameter. The two horizontal lines above and below represent quartiles. *P* < 0.05 was considered statistical significance.
