# Supplementary material for: The feasibility of small-caliber veins for autogenous arteriovenous fistula creation: A single-center retrospective study
Source: Front Cardiovasc Med. 2023 Jan 26;10:1070084. doi: 10.3389/fcvm.2023.1070084 (PMC9909423; doi:10.3389/fcvm.2023.1070084)
Supplement: Supplementary file 1 [file Table_1.DOCX]

Table S1: Preoperative vessels diameter distribution

|  | Vein diameter | Artery diameter |
| --- | --- | --- |
| Minimum (mm) | 0.70 | 1.10 |
| Maximum (mm) | 4.10 | 3.90 |
| 25th quartile (mm) | 1.10 | 1.83 |
| Median (mm) | 1.50 | 2.10 |
| 75th quartile (mm) | 2.00 | 2.40 |
| Mean (mm) | 1.71 | 2.17 |
| Standard deviation (mm) | 0.75 | 0.54 |
| <1mm | 3/106 (2.83%) | 0/106 (0.00%) |
| ≥1mm & <1.5mm | 49/106 (46.23%) | 6/106 (5.66%) |
| ≥1.5mm & <2mm | 18/106 (16.98%) | 26/106 (24.53%) |
| ≥2mm & <2.5mm | 18/106 (16.98%) | 52/106 (49.06%) |
| ≥2.5mm | 18/106 (16.98%) | 22/106 (20.75%) |

Table S2: Sensitivity and specificity of the ROC curve with different thresholds

| Thresholds | Sensitivity | Specificity |
| --- | --- | --- |
| 0.75 | 0.989 | 0.000 |
| 0.85 | 0.978 | 0.000 |
| 0.95 | 0.967 | 0.000 |
| 1.05 | 0.835 | 0.467 |
| 1.15 | 0.769 | 0.533 |
| 1.25 | 0.725 | 0.667 |
| 1.35 | 0.637 | 0.800 |
| 1.45 | 0.560 | 0.800 |
| 1.55 | 0.495 | 0.867 |
| 1.65 | 0.462 | 0.867 |
| 1.75 | 0.407 | 0.867 |
| 1.85 | 0.396 | 0.867 |
| 1.95 | 0.374 | 0.867 |
| 2.05 | 0.275 | 0.933 |
| 2.15 | 0.242 | 0.933 |
| 2.25 | 0.209 | 0.933 |
| 2.35 | 0.198 | 0.933 |
| 2.45 | 0.187 | 0.933 |
| 2.55 | 0.154 | 0.933 |
| 2.65 | 0.143 | 0.933 |
| 2.75 | 0.121 | 0.933 |
| 2.9 | 0.110 | 0.933 |
| 3.05 | 0.088 | 1.000 |
| 3.15 | 0.055 | 1.000 |
| 3.3 | 0.044 | 1.000 |
| 3.65 | 0.033 | 1.000 |
| 4 | 0.011 | 1.000 |

Table S3: Logistic regression analysis of risk factors for additional intervention in 106 cases with preoperative ultrasound data.

| Variable | No additional intervention | Additional intervention | OR | 95%CI | P value |
| --- | --- | --- | --- | --- | --- |
| Male | 56/88 | 8/18 | 2.188 | 0.79-6.28 | 0.135 |
| Female | 32/88 | 10/18 |  |  |  |
| Mean age (years old) | 52.70±15.86 | 53.87±15.92 | 1.023 | 0.99-1.06 | 0.173 |
| Hypertension | 82/88 | 16/18 | 0.585 | 0.12-4.24 | 0.534 |
| Diabetes | 27/88 | 8/18 | 1.807 | 0.63-5.09 | 0.262 |
| Coronary artery disease | 8/88 | 5/18 | 3.846 | 1.03-13.48 | 0.036 |
| Cerebrovascular disease ^a^ | 5/88 | 3/18 | 3.320 | 0.63-15.06 | 0.125 |
| Mean forearm artery diameter (mm) | 2.19±0.55 | 2.04±0.52 | 0.576 | 0.19-1.52 | 0.293 |
| Mean forearm artery PSV (cm/s) | 72.34±92.72 | 58.78±25.84 | 0.997 | 0.97-1.01 | 0.893 |
| Mean forearm vein diameter (mm) | 1.78±0.78 | 1.34±0.35 | 0.306 | 0.09-0.78 | 0.031 |
| Mean brachial artery diameter (mm) | 4.38±0.78 | 4.22±0.76 | 0.759 | 0.37-1.51 | 0.440 |
| Mean brachial artery PSV (cm/s) | 69.60±20.72 | 66.94±24.43 | 0.994 | 0.97-1.02 | 0.648 |
| Mean brachial artery FV (ml/min) | 70.85±50.47 | 66.62±37.02 | 0.998 | 0.98-1.01 | 0.780 |

OR: odd ratio; CI: confidence interval; PSV: peak systolic velocity; FV: flow volume

Values are presented as mean ± SD or number. P<0.05 was considered statistical significance.

a: cerebrovascular diseases include cerebral infarction, cerebral hemorrhage, transient ischemic attack, cerebral arteriosclerosis, etc.
