# Supplementary figures and images for: The feasibility of small-caliber veins for autogenous arteriovenous fistula creation: A single-center retrospective study
Source: Front Cardiovasc Med. 2023 Jan 26;10:1070084. doi: 10.3389/fcvm.2023.1070084 (PMC9909423; doi:10.3389/fcvm.2023.1070084)

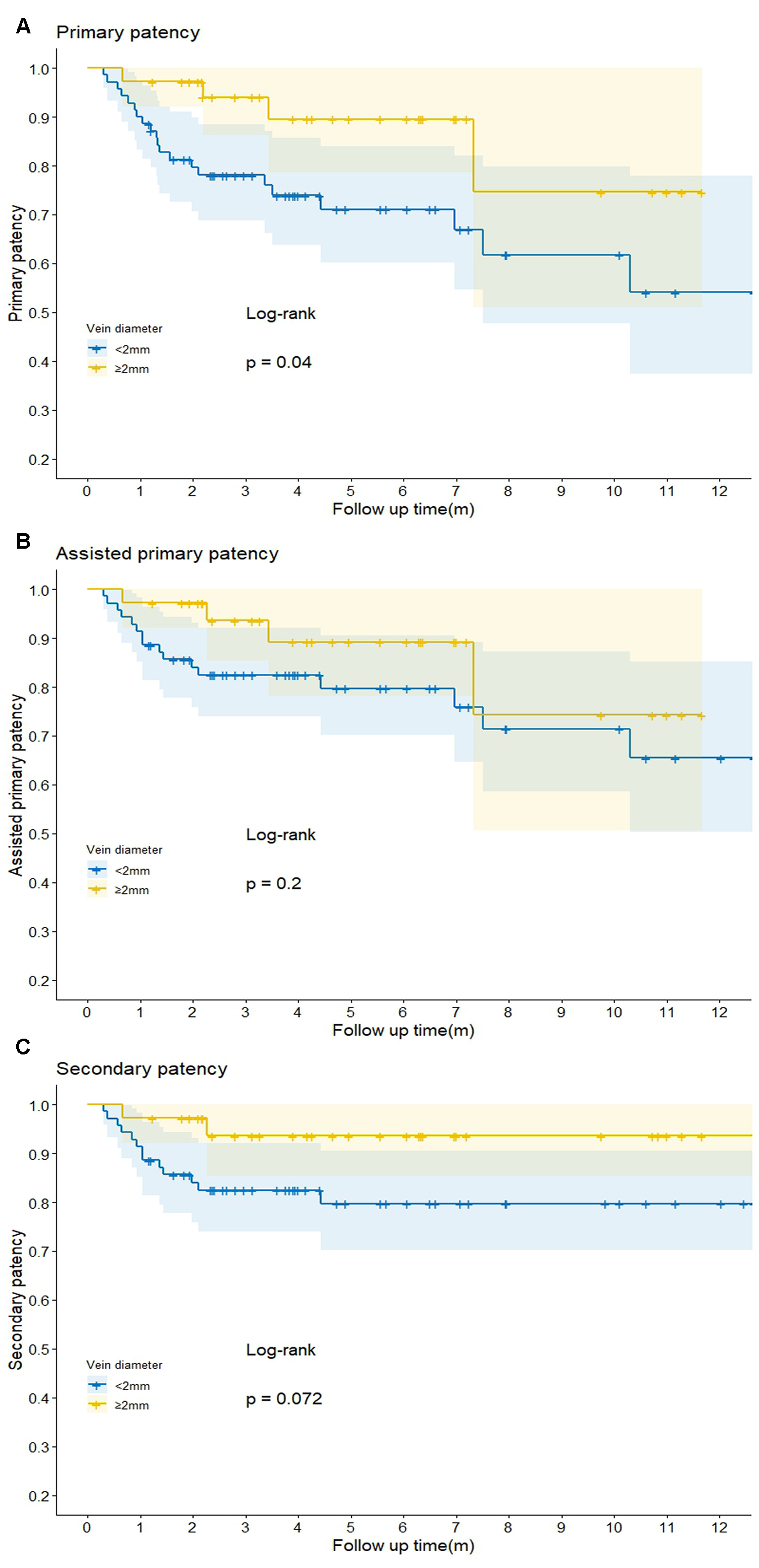

Supplement: SUPPLEMENTARY FIGURE 1 — Comparison of the primary (A), assisted primary (B) and secondary (C) patency rates of the arteriovenous fistula (AVF) in different groups when vein diameter cut-off value was set at 2mm. P < 0.05 was considered statistical significance. [file Image_1.TIF]

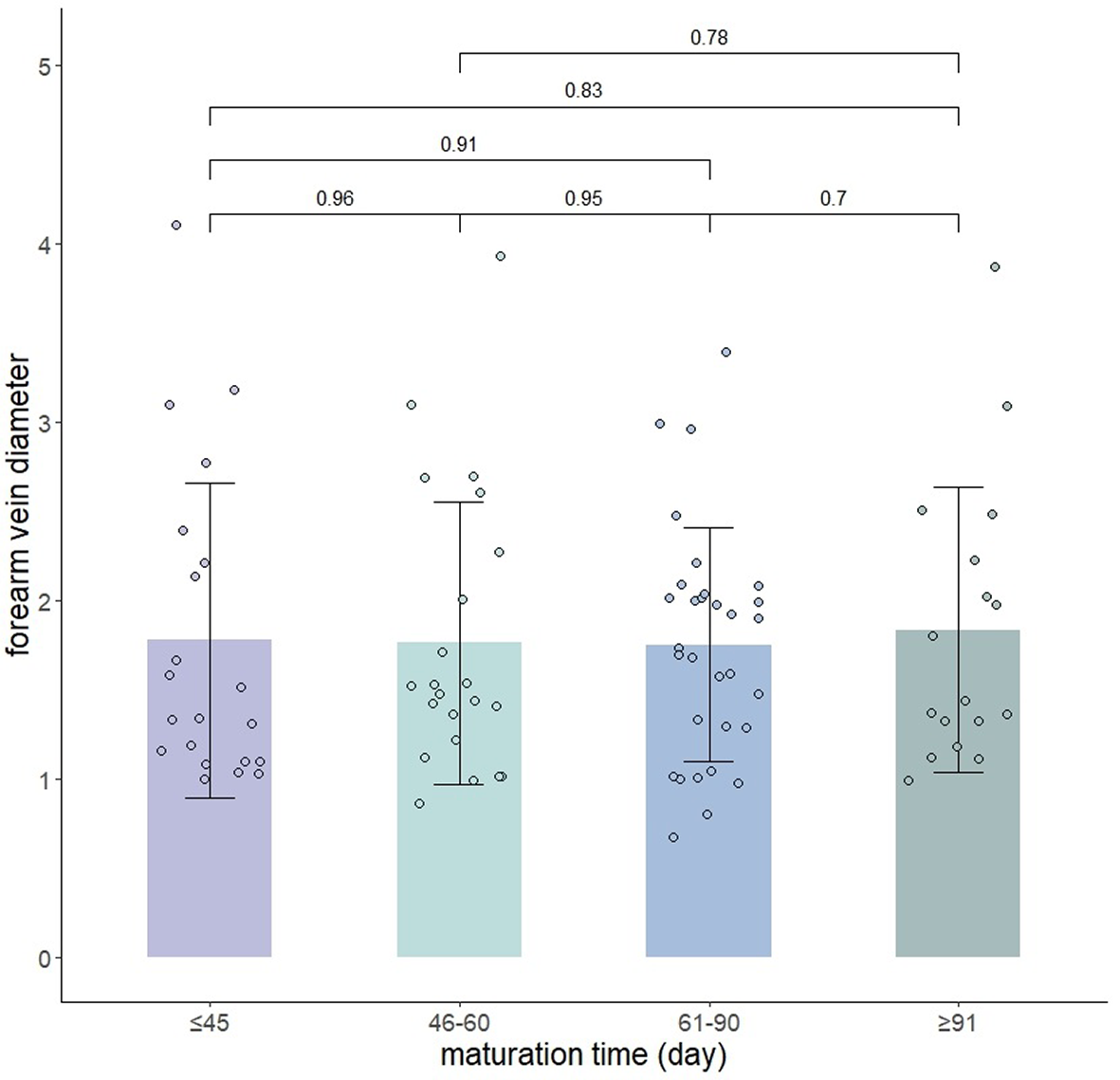

Supplement: SUPPLEMENTARY FIGURE 2 — The different vein diameter distribution grouped by maturation time. Data are expressed as the mean ± SD and compared by two-tailed student’s t test between each two groups. The scattered points represented the specific vein diameter. The two horizontal lines above and below represent quartiles. P < 0.05 was considered statistical significance. [file Image_2.TIF]
